# Ocular Delivery of Therapeutic Agents by Cell-Penetrating Peptides

**DOI:** 10.3390/cells12071071

**Published:** 2023-04-01

**Authors:** Nguyễn Thị Thanh Nhàn, Daniel E. Maidana, Kaori H. Yamada

**Affiliations:** 1 Department of Pharmacology and Regenerative Medicine, University of Illinois College of Medicine, Chicago, IL 60612, USA; nhann@uic.edu; 2 Department of Ophthalmology and Visual Sciences, University of Illinois College of Medicine, Chicago, IL 60612, USA; maidana@uic.edu; 3 Department of Physiology and Biophysics, University of Illinois College of Medicine, Chicago, IL 60612, USA

**Keywords:** CPP, drug delivery, ocular diseases

## Abstract

Cell-penetrating peptides (CPPs) are short peptides with the ability to translocate through the cell membrane to facilitate their cellular uptake. CPPs can be used as drug-delivery systems for molecules that are difficult to uptake. Ocular drug delivery is challenging due to the structural and physiological complexity of the eye. CPPs may be tailored to overcome this challenge, facilitating cellular uptake and delivery to the targeted area. Retinal diseases occur at the posterior pole of the eye; thus, intravitreal injections are needed to deliver drugs at an effective concentration in situ. However, frequent injections have risks of causing vision-threatening complications. Recent investigations have focused on developing long-acting drugs and drug delivery systems to reduce the frequency of injections. In fact, conjugation with CPP could deliver FDA-approved drugs to the back of the eye, as seen by topical application in animal models. This review summarizes recent advances in CPPs, protein/peptide-based drugs for eye diseases, and the use of CPPs for drug delivery based on systematic searches in PubMed and clinical trials. We highlight targeted therapies and explore the potential of CPPs and peptide-based drugs for eye diseases.

## 1. Introduction

**Figure 1:**
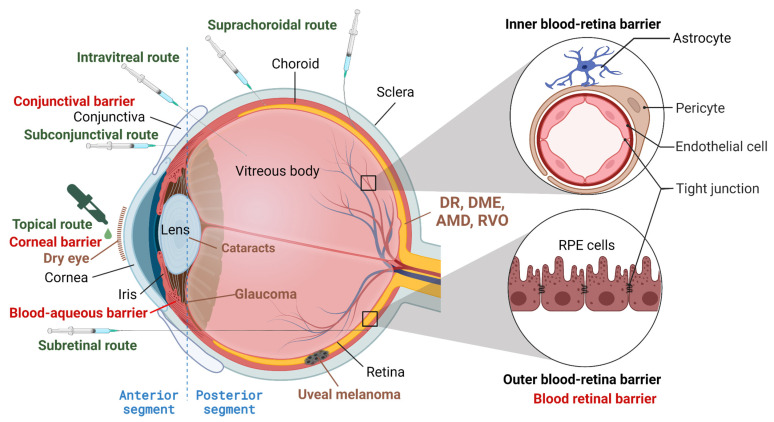
Schemes of the eye structure, common ocular diseases, and ocular drug administration routes. The eye can be divided into an anterior segment and a posterior segment. The anterior segment includes conjunctiva, cornea, anterior and posterior chambers (narrow space behind the iris and in front of the lens). The posterior segment consists of the choroid, retina, and vitreous body. Topical application of ophthalmic formulations is the common route for drug delivery to the anterior segment of the eye for eye diseases that occur at the front of the eye, such as dry eye and glaucoma. Subconjunctival injection can be used for steroid delivery in the setting of ocular inflammation. In contrast, delivery of therapeutic agents to the posterior segment for other eye diseases, including DR, AMD, diabetic macular edema (DME), retinal vein occlusion (RVO), and uveal melanoma, needs invasive administration such as intravitreal, suprachoroidal (microneedle and microcannula), and subretinal injection. Created with BioRender.com.

At least 2.2 billion people worldwide have a vision impairment or blindness [1]. About half of their vision impairment could have been prevented or is yet to be addressed. Thus, ophthalmic medication needs are increasing sharply [1]. The most common sight-threatening ocular diseases are age-related macular degeneration (AMD), cataracts, diabetic retinopathy (DR), diabetic macular edema (DME), retinopathy of prematurity (ROP), dry eye conditions, and glaucoma [2]. In addition, less frequent yet devastating ocular neoplasms, such as uveal melanoma and retinoblastoma, also share the difficulty of drug delivery with other eye diseases. Ocular drug delivery remains challenging due to numerous barriers and mechanisms, such as the blood-aqueous barrier, blood-retina barrier, and the nasolacrimal drainage system, which can significantly affect tissue distribution [3] (Figure 1).

Current proposed administration routes for ocular diseases include subconjunctival [4], intravitreal injection [5,6,7,8,9,10], micro-cannulation or microcatheter [11,12,13], and microneedles [14,15]. However, these therapies are invasive, and most therapies require repeated injections. Less-invasive topical administration, such as contact lenses [16,17,18,19] and conventional topical eye drops [20], have also been developed (Figure 1). Nonetheless, each of these routes has its own advantages and disadvantages. Only a subset of these technologies is efficiently applied in vivo at either pre-clinical or clinical trials and is approved by the FDA in several diseases, as described in Section 5, Section 6, and Section 7 below. Especially for eye diseases occurring at the posterior segment, targeted approaches with antibodies are effectively used in the clinic [21,22,23,24,25,26,27,28,29,30,31,32,33,34,35]. Due to their size, antibody-based drugs are delivered via intravitreal injection. To facilitate the delivery of antibody-based drugs by eyedrops, conjugation of the antibodies with CPP was tested in mouse models and showed encouraging results [36]. As described in Section 3 and Section 4 below, the advantage of using protein/antibody-based drugs is the specificity of their targets. The advantage of using CPPs is the capability of delivering such drugs as non-invasive eye drops.

The delivery of drugs can be influenced by several factors: (i) the transport efficiency in different cells; (ii) rapid endosomal release; (iii) ability to reach the target; (iv) activity at low doses; (v) lack of toxicity; and (vi) facility of therapeutic application [37]. Considering the distribution of drugs, small molecules can easily distribute through the vitreous, while macromolecules are restricted by the barriers described in Figure 1. Moreover, small molecules can bear off-target effects in the eye, leading to lower efficiency and higher toxicity. Therefore, development of technologies optimal for macromolecule delivery, such as proteins and peptides, which target the biologically relevant targets within the eyes, is needed. Cell-penetrating peptides (CPPs), also known as protein-transduction domains (PTDs), can cross the membrane and transport membrane-impermeable cargoes (including small molecules, peptides, proteins, nucleic acids, liposomes, and nanoparticles) into the cells [37]. In recent decades, CPPs have been rising as a potential drug delivery system, especially for the delivery of peptides and proteins in treatment therapies [38,39]. In particular, CPP-conjugated drugs can be administered as eye drops instead of intravitreal injections in ocular diseases [40].

In this review, we first briefly describe the strategies for cell penetration. Then, we focus on the strategies for using CPPs to deliver peptides/proteins in eye diseases and the examples of successfully used peptide/protein drugs for ocular diseases. For writing this review, we searched the literature in PubMed from the initial discovery of CPPs in 1988 to current technological development. We used search terms such as peptide, CPP, cell-penetrating peptide, each name of the eye diseases, and their combinations. For the FDA-approved drugs and drugs in clinical trials, we searched drugs on ClinicalTrials.gov, with search terms such as age-related macular degeneration, diabetic retinopathy, and uveal melanoma. Among the list of agents in clinical trials, we selected peptide or protein-based drugs to describe in this review article.

## 2. The Strategies for Cell Penetration

CPPs consist of less than 30 amino acid residues, which are rich in basic amino acids such as arginine and lysine. CPPs are able to transport numerous cargoes across the cellular membranes and remain in an intact functional form. CPPs were first designed to mimic the natural penetration domain from viruses (TAT) [41], penetratin [42], Pvec [43], etc., which can translocate through the cellular membrane [44]. After that, rational designs for synthetic CPPs were developed. Although the detailed molecular mechanism of cell penetration is still to be elucidated, technologies improving cell penetration have been established. Cationic and amphipathic structures are the main consideration in designing CPPs. Besides the sequence and structure of peptides, other properties, such as internalization efficiency, endosomal escape mechanism, stability, and toxicity, are also crucial in designing CPPs [45].

### 2.1. Cationic CPPs

The cationic class CPPs comprise peptides with highly positive net charges. Most of the conventional CPPs contain cationic charges, such as polyarginine and polylysine. The TAT peptide (GRKKRRQRRRPQ) is the first CPP discovered from the transactivator of the transcription (TAT) of human immunodeficiency virus, and TAT peptide contains six arginine and two lysine residues [41,46]. In addition, non-natural and/or synthetic CPPs containing the guanidinium group or other positively charged residues have also been developed. Synthetic CPPs are beneficial because they allow for the controlled introduction of various chemical entities [47,48,49].

The positive charge of cationic peptides is attracted to the negative charge on the surface of cell membranes, facilitating the interaction of CPPs to the cell membrane to initiate translocation [48,50,51]. Since polyarginine showed a much higher uptake effect compared with others [52], polyarginine is the most studied CPP. However, the mechanisms by which cationic CPPs translocate into the cellular membrane are complicated, with multiple steps, and thus, are somehow controversial. The arginine-rich CPPs were known to be internalized by lipid raft-dependent micropinocytosis, independent of caveolar- and clathrin-mediated endocytosis and phagocytosis [53]. Other groups provided evidence that arginine-rich CPPs directly pass through the membrane via a temporary pore [54,55,56]. Recently, it was reported that arginine-rich CPPs passively enter vesicles and live cells by inducing membrane multilamellarity and fusion [57].

Cationic CPP strategies have been developed for decades for pre-clinical and clinical applications. The most applicable cationic CPPs are TAT and low molecular weight protamine (LMWP). XG-102/AM-111/D-JNKI-1, a TAT-conjugated JNK inhibitor, showed efficacy in suppressing postoperative ocular inflammation in human patients [58]. XG-102/AM-111 has been applied for acute inner ear hearing loss [59]. LMWP consists of 10 arginine residues in its structure (VSRRRRRRGGRRRR) and is a non-toxic CPP for intracellular protein and gene delivery [60]. LMWP conjugation, with insulin, enhanced intestinal absorption of orally administrated insulin in animal models [61]. When delivering siRNA into breast cancer cells to suppress tumor growth and metastasis, conjugation with LMWP facilitated drug delivery of siRNA in vitro [62]. The LMWP-conjugated nanoparticle was used to enhance drug delivery of paclitaxel into s.c. tumor in mice [63] and the delivery of doxorubicin to overcome drug-resistant breast cancer in mice [64]. In addition, LMWP-conjugated nanoparticles greatly facilitated nose-to-brain drug delivery in mice [65]. Finally, studies have highlighted that nanoparticle composition may induce retinal toxicity in vitro, requiring further characterization of these CPP conjugates.

### 2.2. Amphiphilic Peptides

Amphipathic CPPs contain both hydrophilic and hydrophobic residues of amino acids. Besides dominant amino acids, lysine and arginine, amphipathic CPPs are also rich in hydrophobic residues, such as valine, leucine, isoleucine, and alanine [39]. The amphipathic CPPs can be classified into primary structure, secondary structure, or proline-rich CPPs [39,66,67]. Peptides assembled sequentially by a domain of hydrophobic residues, with a domain of hydrophilic residues, are primary amphipathic peptides. For instance, MPG (GALFLGFLGAAGSTMGAWSQPKKKRKV) and Pep-1 (KETWWETWWTEWSQPKKKRKV) are primary amphipathic peptides [68]. Secondary amphipathic peptides are generated by the conformational state that allows positioning of all hydrophobic resides to one face and hydrophilic residues on opposite sides of the molecule, e.g., MAP (KLALKLALKALKAALKLA) [69] and Transportan (GWTLNSAGYLLGKINLKALAALAKKIL) [70]. Proline-rich is a special class that contains a proline pyrrolidine template such as Bac7 (RRIRPRPPRLPRPRPRPLPFPRPG) [71,72].

Since the cellular membrane is composed of an amphipathic bilayer, it is believed that amphiphilic CPPs first interact with the cellular membrane on their hydrophilic face and then penetrate the hydrophobic interior of the cell membrane before delivery of their load to the cytosol. During the cellular translocation process, amphiphilic molecules tend to interact in the aqueous solution so that their nonpolar fragments interact with other nonpolar groups, keeping their polar groups in contact with the aqueous phase [73].

Recently, a combined computational design approach has been developed, which provides scanning of naturally occurring protein sequences for CPP fragments toward the identification of potential amphiphilic peptides [74]. This method allows for shortening the screening step of CPP design. Amphiphilic CPPs have been reported as a carrier for delivery of proteins and CRISPR-associated nucleases to airway epithelia [75].

### 2.3. Hydrophobic Peptides

Hydrophobic CPPs mainly contain nonpolar residues with a low net charge, such as C105Y (CSIPPEVKFNKPFVYLI) or PFVYLI [76]. This class of CPPs is less common and more poorly studied than cationic and amphiphilic classes. The internalization of hydrophobic CPPs is known to be facilitated by their high-affinity hydrophobic motif with the hydrophobic domains of cellular membranes [39]. It has been suggested that hydrophobic CPPs translocate across membranes in an energy-independent manner [77].

### 2.4. Others

The sequence and structure of peptides are important for cellular uptake, but other properties, including the internalization efficiency, endosomal escape mechanism, stability, and toxicity, are also crucial in designing a CPP. Thiol-containing peptides have been studied to improve cellular uptake [78,79,80]. Thiol-containing peptides can cross-react with cell surface thiols to be trapped at the membrane or further internalization [79]. Simply adding a Thiol-tag to a CPP significantly improved the cellular uptake of enzymes, nanobodies, and full-length immunoglobulin-G antibodies [80]. Another strategy is combining CPPs with endosomolytic peptides, such as endosomal leakage domains, which bind and transiently destabilize endosomal membranes, helping CPPs avoid endosomal entrapment [81,82,83].

Although there have been many suggestions to improve cellular uptakes of CPPs, it depends on each specific CPP template and modifications. For example, cationic and hydrophobic properties are the key drivers of cellular uptake, but excess positive charge and hydrophobicity at isolated amino acid positions can trigger membrane lysis [84]. In addition, depending on the cargo or cell type, one strategy can be more advanced than the other. For instance, it was reported that amphipathic CPP is a more suitable carrier moiety than cationic CPP for the delivery of siRNA polyplex [85].

## 3. CPP Is Promising for Ocular Drug Delivery

Despite the increasing number of effective therapeutics for eye diseases, ocular drug delivery has always been challenging due to numerous anatomical and physiological barriers (see Figure 1). Topical application of ophthalmic formulations is the common route for drug delivery to the anterior segment of the eye for eye diseases that occur at the front of the eye, such as dry eye, inflammation (anterior uveitis), and glaucoma. In contrast, it is still challenging to deliver therapeutic agents to the posterior segment of the eyes for other eye disorders at the back of the eye, including DR, AMD, diabetic macular edema (DME), retinopathy of prematurity (ROP), retinal vein occlusion (RVO), and posterior uveitis [86,87]. Posterior segment diseases are treated by local injections, such as periocular, intravitreal, suprachoroidal, and subretinal (Figure 1). Conventional ophthalmic drugs for treating posterior segment disorders are intravitreal injections of anti-vascular endothelial growth factor (VEGF) agents [30,32,33,88,89,90,91,92,93]. Bevacizumab (Avastin) and ranibizumab (Lucentis) are a monoclonal antibody and an antibody fragment, respectively, which bind to VEGF-A to inhibit VEGF signaling in choroidal neovascularization (CNV) [93]. Aflibercept (Eylea, VEGF-Trap) is a recombinant protein derived from the extracellular domains of VEGFR1 and VEGFR2 [92]; thus, aflibercept interacts with and inhibits signaling from VEGF family proteins, including VEGF-A, -B, -C, -D, and placenta growth factor (PGF). VEGF blockage serves as the gold standard for treating wet AMD, DR, and DME, as high VEGF is a hallmark of these diseases [91]. However, the conspicuous limitation of this therapy is the invasion and repetitive injection. Therefore, non-invasive, effective, and innovative therapies are needed.

CPPs appear as possible enhancing strategies for the noninvasive delivery of potent therapeutic agents because they can be used as eye drops. Drug delivery of anti-VEGF (bevacizumab and ranibizumab) to the posterior segment using CPPs was tested in the laser-induced CNV model, a well-established wet AMD model [36]. Eyedrops of bevacizumab and ranibizumab, conjugated with CPPs, were as efficacious as a single intravitreal injection of anti-VEGF in reducing areas of CNV in vivo [36]. If successfully developed, CPP-conjugation, with currently injectable drugs, can make them topically applicable as eye drops to reduce the burden for patients who currently need to visit the doctor’s office for monthly injections. Further testing for safety, toxicity, and pharmacokinetics with larger animals would be needed before applying it to human patients.

## 4. Advantages of Peptide-Based Therapeutics

In previous decades, the primary class of therapeutics is small molecules, as they can rapidly diffuse through biological fluids, across biological barriers, and through cell membranes, which allow small molecules to approach and interact with most tissues and cell types in the body [94]. However, due to the free diffusion, they usually cause off-target, leading to high toxicity. In addition, they limit the application of less soluble molecules [95]. Together with small molecule-based therapeutics, advanced biomedical sciences provided a remarkable expansion of peptide- and protein-based therapeutics. These large molecules offer many advantages compared to small-molecule drugs. Their large size and diverse structures increase specificity, resulting in higher potency and reduced toxicity. Peptides and proteins also provide a broad range of targets without a limitation of types and structure [96,97,98]. Despite the similar mechanisms, compared to proteins and antibodies, peptides have more benefits in cell penetration, immune escape, and production costs because of their smaller size [99]. Although therapeutic peptides face two major challenges—membrane impermeability and poor in vivo stability—novel approaches are showing potential to improve these drawbacks through chemical or biological peptide synthesis and sequence modification [99,100]. Peptide optimization strategies, including chemical, backbone, and sidechain modifications, have enhanced permeability, reducing proteolysis and renal clearance and prolonging half-life [99,101]. Small peptide kinesin-derived angiogenesis inhibitor (KAI) was able to penetrate through the cell membrane using cationic residues and inhibited metastasis in the cancer model and angiogenesis and vascular leakage in the wet AMD model in vivo [102].

## 5. Successfully Used Peptide/Protein Drugs for Eye Diseases

Protein- and/or peptide-based therapeutics have emerged to address multiple ocular diseases. High expression of VEGF in wet AMD, DR, ROP, and DME causes leakiness of ocular vessels and unwanted angiogenesis. Thus, the anti-VEGF strategy was successfully developed. In addition to Lucentis and Eylea, mentioned above, Brolucizumab (Beovu), Byooviz, and Faricimab (Vabysmo) are FDA-approved protein drugs for eye diseases (Table 1).

Beovu/Brolucizumab (Novartis, Basel, Switzerland) is an antibody fragment for VEGF-A, approved for wet AMD in 2019 [21,22]. However, Beovu has been found to have an unacceptable safety profile with a risk of inflammation-induced retinal vascular occlusions (RVO) and visual acuity loss [23].

Byooviz/Ranibizumab-nuna/SB11 (Biogen/Samsung Bioepis, Incheon, South Korea) is a biosimilar to Lucenstis, approved for wet AMD, DME, RVO, and myopic choroidal neovascularization (mCNV) [24,25].

Vabysmo/faricimab (Roche/Genentech, South San Francisco, CA, USA) is a dual-blocking antibody for both VEGF-A and Ang2 [26]. Ang2 (Angpt2) induces sprouting angiogenesis and vascular leakage by interacting with Tie2. Thus, blocking both Ang2 and VEGF-A is more effective than a single blockade for treating wet AMD and DME [27,28]. Faricimab was approved for wet AMD and DME in 2022.

One of the remaining challenges is the burden of frequent intravitreal injections. Since all approved therapies are proteins or antibodies, they cannot be used as eye drops. Susvimo (Genentech) was developed to reduce the burden of frequent office visits for intravitreal injections. Susvimo is a refillable port delivery system that slowly releases ranibizumab into the vitreous and was approved for wet AMD in 2021 [29]. Long-acting anti-VEGF therapies are also tested in clinical trials, as described in the next section.

## 6. Peptide/Protein Drugs for Eye Diseases in Clinical Trials

In this section, we review the potential peptide/protein-based therapies for eye diseases in clinical trials (Table 2). Based on the success of anti-VEGF antibodies in wet AMD, targeted therapies using antibodies are popular for drug development for eye diseases. To improve drug delivery, smaller antigen-binding fragments are preferred to full-length antibodies.

### 6.1. Anti-VEGF Therapy

Since intravitreal injection of anti-VEGF drugs is the gold standard for therapies of eye diseases, such as wet AMD, DME, and DR, biosimilars for successful anti-VEGF drugs (ranibizumab, bevacizumab, and aflibercept) have been developed and tested in clinical trials [135,136]. CKD-701 (Chong Kun Dang Pharmaceutical, Seoul, South Korea) is biosimilar for ranibizumab. Phase III (NCT04857177), to examine the efficacy of CKD-701 compared with ranibizumab for wet AMD patients, was successfully completed and showed improvement and maintenance of visual outcome through PRN (as needed) regimen [103]. HLX04-O (Shanghai Henlius Biotech, Shanghai, China) is biosimilar for bevacizumab. Phase I/II (NCT04993352) showed efficacy of HLX04-O to improve visual acuity for wet AMD patients [104] and proceeded for Phase III trials (NCT05003245, NCT04740671) to compare the efficacy with ranibizumab. Similar to bevacizumab, ranibizumab, and aflibercept, these biosimilars also need repeated intravitreal injections.

Conbercept/Lumitin/KH902 (Chengdu Kanghong Biotech Company, Chengdu, China) is a new anti-VEGF antibody, approved by China State FDA for the treatment of wet AMD in 2013 after completion of the Phase II AURORA trial (NCT01157715) [105] and Phase III PHOENIX trial (NCT01436864) [106]. Phase II FALCON (NCT01809236) and Phase III BRAVE (NCT03108352) [108] for macular edema secondary to retinal vein occlusion (RVO), were also completed in China. Conbercept is currently used for wet AMD, DME, and RVO. However, worldwide Phase III clinical trials PANDA-1 and PANDA-2 (NCT03630952 and NCT03577899) were terminated because the desired primary endpoint was not met. Conbercept was also tested for uveal melanoma after plaque radiotherapy and showed partly relieved retinal vascular damage in response to radiation therapy, but it may not provide long-term positive functional outcomes [137].

In addition to VEGF-A, VEGF-C and -D also contribute to the disease progression for wet AMD. Thus, combination therapy of OPT-302 (targeting VEGF-C and -D; Opthea, Victoria, Australia) with ranibizumab (targeting VEGF-A) was tested in the Phase IIb trial for wet AMD (NCT03345082), showing greater improvement in visual acuity than ranibizumab monotherapy, and proceeded to Phase III (NCT04757636 and NCT04757610) [109,110].

To reduce the burden of frequent injections, long-acting drugs are developed by using engineered antibody-mimetic proteins. DARPin (designed ankyrin repeat proteins) is a class of small, highly stable, engineered binding proteins containing ankyrin repeat domains. Abicipar pegor (Allergan, an AbbVie Company, Irvine, CA, USA) is a DARPin molecule. Abicipar binds all VEGF-A isoforms, similar to ranibizumab, with a higher affinity and longer intraocular half-life than ranibizumab [138,139]. In fact, Phase III randomized, controlled studies CEDAR and SEQUOIA (NCT02462928, NCT02462486), for treatment of wet AMD, showed that quarterly (every 12 weeks) injection of Abicipar is non-inferior to monthly ranibizumab [111]. Abicipar was thought to reduce the frequency of injections. However, Abicipar was not approved for the treatment of wet AMD due to the rate of intraocular inflammation observed after the injection of Abicipar [140].

MP0112 (Allergan) is another DARPin, a long-acting VEGF inhibitor, showing promising results in Phase I (NCT01086761) [112].

### 6.2. Anti-Ang2

Targeting more than one pathway is also a promising strategy to ameliorate symptoms induced by multiple pathways. A successful example is FDA-approved faricimab (Genentech), a fusion protein targeting both VEGF and Ang2 [27,28]. With a similar strategy, a combination of anti-VEGF and anti-Ang2 was tested. Anti-Ang2 monoclonal antibody, nesvacumab/REGN910/SAR307746 (Regeneron Pharmaceuticals, Tarrytown, NY, USA), was originally developed for cancer therapy (NCT01271972) [141]. The combination of nesvacumab and aflibercept injection was not better than aflibercept single treatment for DME in Phase II (NCT02712008) [113]; thus, the company suspended a move to Phase III. AM712/ASKG712 (AffaMed Therapeutic, Shanghai, China) is also designed to inhibit both VEGF and Ang2, and Phase I trials (NCT05345769, NCT05456828) are recruiting.

### 6.3. Anti-PDGF Therapy

Despite the initial effectiveness of anti-VEGF therapy, visual acuity declines beyond baseline levels in most patients during the first four years of treatment [142,143,144]. In the limitation of anti-VEGF therapy, pericytes are thought to play an important role [116]. Pericytes cover the endothelial monolayer of blood vessels, provide endothelial cells with growth factors, and protect endothelial cells from anti-VEGF therapy. Pericytes recruitment, maturation, and survival are mediated by platelet-derived growth factor (PDGF). Thus, dual blocking of VEGF and PDGF pathways is considered. Rinucumab/REGN2176 (Regeneron Pharmaceuticals) is an anti-PDGFRβ, co-formulated with aflibercept. After successful completion of the Phase I safety study (NCT02061865), rinucumab was tested in Phase II CAPELLA (NCT02418754) to compare the efficacy with aflibercept alone. However, the Phase II trial was terminated as intravitreal injection of rinucumab (anti-PDGFRβ + aflibercept) did not show any additional efficacy over aflibercept alone in wet AMD patients [115].

Fovista/E10030/pegpleranib (Ophthotech Corporation, Prinston, NJ, USA) is an anti-PDGF antibody tested as a combination therapy with ranibizumab and showed superior efficacy over ranibizumab alone in a Phase II trial for wet AMD patients (NCT01089517) [116]. However, international, multicenter, randomized, double-masked, controlled Phase III clinical trials OPH1002 and OPH1003 (NCT01944839, NCT01940900) were terminated as a combination of Fovista and ranibizumab was not better than ranibizumab monotherapy [115].

### 6.4. Anti-Inflammation Therapy

The approved therapies (Table 1) are for angiogenesis-related eye diseases, such as wet AMD, DR, DME, mCNV, and RVO. There is no approved therapy for dry AMD [145]. Among AMD, the exudative neovascular type of AMD is called wet AMD, whereas the non-exudative type of AMD is dry AMD. At the early stage of AMD, accumulation of drusen is observed in Bruch’s membrane [146]. Complement factors found in drusen induce inflammatory response and worsen AMD. Thus, therapies targeting the complement system and inflammation have been developed.

Tesidolumab/LFG316 (Novartis) is an antibody that prevents the cleavage of C5. The safety and efficacy of LFG316 was tested for dry AMD (NCT01527500) and wet AMD (NCT01535950, NCT01624636), showing no efficacy [117].

Phase III clinical trials of anti-factor D Lampalizumab (Genentech) (NCT02247531, NCT02247479) [118] and the Phase II trial of systemic complement C5 inhibition with eculizumab (Alexion Pharmaceuticals, Boston, MA, USA) (NCT00935883) [119] failed to show a treatment benefit.

GEM103 (Gemini Therapeutics, Wayland, MA, USA) is a recombinant human complement factor H, tested for dry AMD in a Phase I clinical trial (NCT04246866). Intravitreal injection of GEM103, up to 500 µg/eye, was well tolerated [120]; however, Phase II clinical trials (NCT04643886, NCT04684394) were terminated due to lack of treatment benefit.

Amyloid β is also found in drusen. GSK933776 (GlaxoSmithKline, Middlesex, United Kingdom), an anti-amyloid β antibody, was first developed for Alzheimer’s Disease and was tested in a Phase II trial for dry AMD (NCT01342926) [121] but failed to show a treatment benefit [123]. RN6G/PF-43829223 (Pfizer, New York, NY, USA) is also an anti-amyloid β antibody. However, the Phase II trial for dry AMD (NCT01577381) was terminated for lack of efficacy.

Success of the therapy, targeting the complement system, was finally obtained by using a peptide. Pegcetacoplan/APL-2 (Apellis Pharmaceuticals, Waltham, MA, USA) is a PEGylated pentadecapeptide, sold under the brand name Empaveli, for treatment of paroxysmal nocturnal hemoglobinuria [147]. Pegcetacoplan binds to complement protein C3 and its activation fragment C3b with high affinity, thereby regulating the cleavage of C3 and the generation of downstream effectors of complement activation. Pegcetacoplan completed two Phase III trials, DERBY and OAKS, for dry AMD (NCT03525613, NCT03525600), showing a reduction in the rate of geographic atrophy lesion growth compared to sham injection [125].

Another example of success is the dual-blocking strategy. Efdamrofusp alpha/IBI302 (Innovent Biologics, Jiangsu, China) is a bispecific decoy receptor fusion protein for VEGF and complement [126,148]. A randomized, open-label Phase Ib study (NCT04370379) showed that monthly intravitreal injection of efdamrofusp alpha, dosed up to 4 mg, was well tolerated in wet AMD patients, with similar improvement of visual acuity compared to aflibercept [127].

### 6.5. Drugs Targeting Other Molecules

Despite standardized anti-VEGF therapy, there are still non-responders, ~30%, among wet AMD patients. Thus, drugs targeting other molecules are developed as a single or combination therapy with anti-VEGF therapies.

RC28E (RemeGen, Yantai Shandong, China) is a dual decoy receptor for VEGF and bFGF and was tested in Phase I/II clinical trial (NCT04270669) for wet AMD and Phase II clinical trials (NCT04782128, NCT04782115) for DR and DME.

An antibody for endoglin, DE-122/Carotuximab/TRC105 (Santen, Osaka, Japan), was well tolerated with no serious side effects in Phase I/II for AMD (NCT02555306) [129]; however, it failed to show treatment benefits in Phase II (NCT03211234).

Phase II NEXUS clinical trial for iSONEP, S1P antibody, for wet AMD (NCT01414153) failed, as it did not meet its primary or key secondary endpoints [130].

DS-7080a (Daiichi Sankyo, Tokyo, Japan) is an antibody against ROBO4, which has an anti-angiogenic effect [149]. DS-7080a is tested for wet AMD and DME in Phase I (NCT02530918).

High expression of tissue factor (TF) was found in choroidal neovascularization in wet AMD. HI-con1 is an antibody-like molecule targeted against TF, composed of two human factor VII. HI-con1 was tested for Phase I/II and Phase II trials for wet AMD (NCT01485588, NCT02358889), showing promising results [132].

Another promising drug is a peptide. ALG-1001/Risuteganib/Luminate (Allegro Ophthalmics, San Juan Capistrano, CA, USA) is an anti-integrin oligopeptide for dry AMD and DME [150]. Risuteganib inhibits function of four different integrin heterodimers (αVβ3, αVβ5, α5β1, and αMβ2) involved in the pathogenesis of AMD and DME. Risuteganib was tested in Phase I/II (NCT01482871) and Phase II (NCT02348918) for DME and Phase I/II (NCT01749891) and Phase II (NCT03626636) for dry AMD.

Peptide-drug was also developed for ocular inflammation after surgery. XG-102/AM-111/brimapitide/D-JNKI-1 is a TAT-coupled dextrogyre peptide containing a 20-aa sequence of the JNK-binding domain, combined with a 10-aa TAT sequence of the TAT protein that selectively inhibits the c-Jun N-terminal kinase in vitro [151]. In Phase I clinical study in treating postoperative ocular inflammation, a single subconjunctival injection of XG-102 at the end of ocular surgery is non-inferior to dexamethasone eye drops [58]. Subconjunctival injection (Figure 1) could be considered one of the less invasive and easily accessible routes for drug delivery to both anterior and posterior segments of the eye [152].

The last example is an antibody for IL-1β, tested for dry eye. Canakinumab/ACZ885 (Novartis) is an anti-IL-1β antibody, sold as ILARIS, for the treatment of Still’s Disease, idiopathic arthritis, and periodic fever syndromes. Canakinumab systemic administration did not ameliorate the severity of dry eye (NCT01250171) [134].

## 7. Pre-Clinical Peptide Drug Development

In addition to the aforementioned protein/peptide drugs in the clinic and clinical trials, there are many peptide-based drugs in pre-clinical studies.

To provide the alternative therapy for the non-responders for anti-VEGF therapies, a new approach was used to target VEGFR2 trafficking. Since VEGFR2 needs to be exposed to the cell surface to receive VEGF, inhibition of VEGFR2 trafficking to the cell surface can reduce VEGF/VEGFR2 signaling [153]. KIF13B is a kinesin family protein, mediating VEGFR2 trafficking to the cell surface [153]. To inhibit the interaction between KIF13B and VEGFR2, KAI (a kinesin-derived angiogenesis inhibitor), a 23-amino acid peptide (aa 1238–1260) of human KIF13B, was designed from the minimum binding site to VEGFR2. KAI prevents interaction of VEGFR2 with KIF13B, thus inhibiting the trafficking of VEGFR2 to the EC plasmalemma [154]. KAI peptide was designed as a cationic CPP facilitating cellular uptake. Topically applied KAI as an eyedrop was successfully delivered to the back of mouse eyes and reduced the disease progression in laser-induced CNV, an animal model of wet AMD [155].

CPPs can also be used as a drug delivery strategy. Currently, protein/antibody-based drugs need intravitreal injections. Conjugating CPP with bevacizumab and ranibizumab could successfully deliver these drugs to the back of the eyes and showed efficacy in reducing neovascularization in animal models [36].

Peptide for ocular delivery (POD), a 3.5 kD-peptide GGG(ARKKAAKA)_4_, was able to deliver both small and large molecules into the back of the eyes. POD is believed to resemble the glycosaminoglycan binding regions of proteins abundantly present in the retina, resulting in protein transduction properties in the eyes. POD was able to enter retinal pigment epithelium (RPE), photoreceptor, ganglion cells, corneal epithelium, sclera, choroid, etc., via topical application in vivo [156,157].

TAT is also popularly used as a drug delivery system. TAT-conjugated aFGF-His (TAT-aFGF-His) exhibited efficient penetration into the retina, following topical administration to the ocular surface. After retinal ischemia-reperfusion injury, retina from TAT-aFGF-His-treated rats showed better-maintained inner retinal layer structure, reduced apoptosis of retinal ganglion cells, and improved retinal function compared to those treated with aFGF-His or PBS [158].

As for CPP delivery of peptides, TAT-μCL, an inhibitory peptide that specifically acts against mitochondrial μ-calpain, was successfully delivered to the back of the eyes of rats and protected photoreceptors in retinal dystrophic rats [159,160]. TAT-μCL is a 23 amino acid, 2857.37 Da peptide, with a 10 amino acid stretch of the μ-calpain inhibitory domain at the carboxy-terminal end, and an additional 13 amino acid fragment of the TAT peptide, conjugated at the amino-terminal end to enhance membrane penetration [159,161]. TAT-μCL CPP was distributed to both anterior and posterior segments of the rat eyes after topical administration, promising a potential strategy for ocular drug delivery.

## 8. Future Perspective

The efficacy of drugs for eye diseases relies heavily on the successful delivery of the drugs to the area of the disease and the retention of the drugs there. CPPs have potential to provide the delivery of drugs even to the back of the eyes. The TAT-conjugated drug, XG-102 [58], is such an example. CPPs can also be conjugated to non-protein/peptide drugs such as siRNA. Bevasiranib is a siRNA for VEGF, tested in a Phase III clinical trial for AMD (NCT00557791). Conjugation of CPPs may improve the efficacy of such drugs.

Designing peptides with drug delivery functions, such as CPPs and inhibitory effects, is also an effective strategy. ALG-1001, pegcetacoplan, and KAI are such examples.

Considering the benefits of targeted therapies and the increasing number of elderly patients with eye diseases, the need to use peptides/proteins for treatments of eye diseases will increase. Further development of therapies will benefit such patients with eye diseases. 

## 9. Conclusions

In this review, we discussed the strategies for designing CPPs, the use of CPPs as drug delivery systems, currently available protein/peptide-based therapies for eye diseases, and drug delivery for eye diseases. Cationic CPP, TAT, is used to deliver the peptide drug XG-102 to reduce ocular inflammation after surgery for cataracts and has been tested in clinical trials. A strategy of CPP conjugation with currently available anti-VEGF drugs was tested in pre-clinical animal models. Peptide-based drugs, risuteganib and pegcetacoplan, are in clinical trials for testing their efficacy in AMD and DME. Other peptide-based drugs, KAI, POD, TAT-aFGF-His, and TAT-μCL, are also in pre-clinical drug development. Improving such strategies by conjugating with less-toxic CPPs instead of TAT and formulating peptide-based drugs with hydrogel to facilitate drug delivery by topical route (eyedrop) would be needed for further development. The potential risk of toxicity on the ocular surface and inflammation should be carefully tested in animal models. While further investigation is required to unlock the full potential of CPPs, new CPP-based approaches can ultimately provide more efficient and less-invasive treatment options for eye diseases.

## Figures and Tables

**Table 1:** FDA-approved therapies for eye diseases.

Generic Name	Brand Name	Format	Company	Year	Conditions	Ref.
Ranibizumab	Lucentis	Anti-VEGF-A antibody	Genentech	2006	Wet AMD, DR, DME, mCNV, RVO	[30,31,32]
Aflibercept	Eylea	Recombinant protein targeting VEGF	Regeneron	2011	Wet AMD, ROP	[33,34,35]
Brolucizumab	Beovu	Anti-VEGF-A antibody	Novartis	2019	Wet AMD	[21,22]
	Susvimo	Ocular implant for ranibizumab	Genentech	2021	Wet AMD	[29]
Ranibizumab-nuna (SB11)	Byooviz	Biosimilar of ranibizumab	Biogen/Samsung Bioepis	2021	Wet AMD, DME, RVO, mCNV	[24,25]
Faricimab	Vabysmo	Ab targeting both VEGF-A and Ang2	Roche/Genentech	2022	Wet AMD, DME	[27,28]

Wet AMD: wet age-related macular degeneration, DR: diabetic retinopathy, DME: diabetic macular edema, mCNV: myopic choroidal neovascularization, RVO: macular edema following retinal vein occlusion, ROP: retinopathy of prematurity.

**Table 2:** Developing peptide/protein-based drugs for eye diseases in clinical trials.

Name	Type	Format	Clinical Trials	Route	Conditions	Ref.
CKD-701	Antibody	Anti-VEGF antibody fragment	Phase 3 (NCT04857177)	IVT	Wet AMD	[103]
HLX04-O	Antibody	Anti-VEGF antibody	Phase 1/2 (NCT04993352), phase 3 (NCT05003245, NCT04740671)	IVT	Wet AMD	[104]
Conbercept/Lumitin/KH902	Protein	A recombinant protein targeting all VEGF isoforms and PlGF	Phase 2 (NCT01157715), phase 3 (NCT01436864), Phase 2 (NCT01809236) and phase 3 (NCT03108352), phase 3 PANDA-1 and PANDA-2 (NCT03630952 and NCT03577899)	IVT	Wet AMD, DME, RVO	[105,106,107,108]
OPT-302	Protein	A recombinant protein targeting VEGF-C and VEGF-D	Phase 2 (NCT03345082), phase 3 (NCT04757636, NCT04757610)	IVT	Wet AMD	[109,110]
Abicipar	Protein	Engineered protein with Ankyrin repeat targeting VEGF	Phase 3 (NCT02462928, NCT02462486)	IVT	Wet AMD	[111]
MP0112	Protein	DARPin, a long-acting VEGF inhibitor	Phase 1 (NCT01086761)	IVT	Wet AMD	[112]
Nesvacumab/REGN910/SAR307746	Antibody	Anti-Ang2	Phase 2 (NCT02712008)	IVT	DME	[113]
AM712/ASKG712	Protein	A bifunctional molecule targeting VEGF and Ang2	Phase 1 (NCT05345769, NCT05456828)	IVT	Wet AMD	[114]
Rinucumab/REGN2176	Antibody	Anti-PDGFRβ	Phase 1 (NCT02061865), phase 2 (NCT02418754)	IVT	Wet AMD	[115]
Fovista/pegpleranib	Antibody	Anti-PDGF	Phase 2 (NCT01089517)	IVT	Wet AMD	[116]
Tesidolumab/LFG316	Antibody	An antibody that prevents the cleavage of C5	Phase 2 trials for wet AMD (NCT01535950, NCT01624636), for dry AMD (NCT01527500)	IVT	Wet AMD, dry AMD	[117]
Lampalizumab	Antibody	Anti-factor D	Phase 3 (NCT02247531, NCT02247479)	IVT	Dry AMD	[118]
Eculizumab	Antibody	Anti- C5	Phase 2 (NCT00935883)	Systemic	Dry AMD	[119]
GEM103	Protein	A recombinant human complement factor H	Phase 1 (NCT04246866), phase 2 (NCT04643886, NCT04684394)		Dry AMD	[120]
GSK933776	Antibody	Anti-amyloid β antibody	Phase 2 (NCT01342926)	IVT	Dry AMD	[121,122,123]
RN6G/PF-43829223	Antibody	Anti-amyloid β antibody	Phase 2 trial for dry AMD (NCT01577381)	IVT	Dry AMD	[124]
Pegcetacoplan/APL-2/Empaveli	Peptide	C3 inhibitor	Phase 3 trials DERBY and OAKS for dry AMD (NCT03525613, NCT03525600)	IVT	Dry AMD	[125]
Efdamrofusp alpha/IBI302	Protein	Bispecific decoy receptor fusion protein for VEGF and complement	Phase 1 (NCT03814291, NCT04370379)	IVT	Wet AMD	[126,127]
RC28-E	Protein	Dual decoy receptor targeting VEGF and bFGF	Phase 1/2 clinical trial (NCT04270669), Phase 2 (NCT04782128, NCT04782115)	IVT	DR, DME, wet AMD	[128]
DE-122/Carotuximab/TRC105	Antibody	An antibody for endoglin	Phase 1/2 for AMD (NCT02555306), phase 2 (NCT03211234).	IVT	AMD	[129]
iSONEP	Protein	Anti-S1P	Phase 2 (NCT01414153)			[130]
DS-7080a	Antibody	Anti-ROBO4	Phase 1 (NCT02530918)	IVT	Wet AMD, DMED	[131]
HI-con1 is an antibody-like molecule targeted against tissue factor (TF), composed of two human factor VII	Antibody	A factor VII-IgGFc chimeric protein targeting tissue factor	Phase 1/2 (NCT01485588), phase 2 (NCT02358889)	IVT	Wet AMD	[132]
ALG-1001/Risuteganib/Luminate	Peptide	Anti-integrin oligopeptide	Phase 2 for dry AMD (NCT03626636), phase 1/2 for AMD (NCT01749891), phase 2 for DME (NCT02348918), phase 2 for vitreomacular adhesion (NCT02153476)	IVT	Dry AMD, wet AMD, DMO, vitreomacular adhesion	[133]
XG-102/AM-111/brimapitide/D-JNKI-1	Peptide	A TAT-coupled JNK inhibitor	Phase 3 (NCT02235272, NCT02508337)	IVT	Postoperative ocular inflammation	[58]
Canakinumab/ACZ885/ILARISs	Antibody	Anti-IL-1β antibody	Phase 2 (NCT01250171)	IVT	Dry AMD	[134]

## Data Availability

Not applicable.
